# DEG 10, an update of the database of essential genes that includes both protein-coding genes and noncoding genomic elements

**DOI:** 10.1093/nar/gkt1131

**Published:** 2013-11-15

**Authors:** Hao Luo, Yan Lin, Feng Gao, Chun-Ting Zhang, Ren Zhang

**Affiliations:** ^1^Department of Physics, Tianjin University, Tianjin 300072, People’s Republic of China and ^2^Center for Molecular Medicine and Genetics, School of Medicine, Wayne State University, Detroit 48201, USA

## Abstract

The combination of high-density transposon-mediated mutagenesis and high-throughput sequencing has led to significant advancements in research on essential genes, resulting in a dramatic increase in the number of identified prokaryotic essential genes under diverse conditions and a revised essential-gene concept that includes all essential genomic elements, rather than focusing on protein-coding genes only. DEG 10, a new release of the Database of Essential Genes (available at http://www.essentialgene.org), has been developed to accommodate these quantitative and qualitative advancements. In addition to increasing the number of bacterial and archaeal essential genes determined by genome-wide gene essentiality screens, DEG 10 also harbors essential noncoding RNAs, promoters, regulatory sequences and replication origins. These essential genomic elements are determined not only *in vitro*, but also *in vivo*, under diverse conditions including those for survival, pathogenesis and antibiotic resistance. We have developed customizable BLAST tools that allow users to perform species- and experiment-specific BLAST searches for a single gene, a list of genes, annotated or unannotated genomes. Therefore, DEG 10 includes essential genomic elements under different conditions in three domains of life, with customizable BLAST tools.

## INTRODUCTION

Delineating a set of essential genomic elements and proteins that make up a living organism helps to understand critical cellular processes that sustain life (1–3). Identification of essential genes is especially useful to studies of synthetic biology (4), which seeks to make an artificial self-sustainable living cell, with addable gene circuitries that encode desirable traits. Bacterial essential genes, because of their lethality phenotype, are attractive drug targets, and this is especially important for those having multidrug resistance (5).

Reverse genetics (from gene disruption to phenotypic characterization) has been extensively used to experimentally determine essential genes. One standard method is to perform targeted mutagenesis in a particular gene of interest. Classical examples include essential gene determination in *Bacillus subtilis* (6) and *Escherichia coli* (7), in which all protein-coding genes are deleted one by one. This method gives a clear-cut answer on gene lethality, but it is labor-intensive, time-consuming and requires detailed genome annotation. Single-gene knockout screens can overlook genes causing synthetic lethality, which refers to lethal phenotypes caused by genetic interactions of genes that are nonessential when deleted separately (3). Indeed, duplicated genes are less likely to be essential than singletons (8). Another method is to construct a random transposon-insertion library, followed by determination of insertion sites by DNA hybridization (9) or microarray (10), which suffers from some shortcomings including missing low-abundance transcripts, low resolution in locating insertion sites, and narrow ranges in counting probe density. An advantage of global transposon mutagenesis is that it can simultaneously identify essential noncoding elements in addition to protein-coding genes.

The combination of high-density transposon-mediated mutagenesis and high-throughput sequencing has resulted in significant advancements in the study of essential genes (11). This method, however, has in fact been gradually developed for more than 10 years. In 1999, Venter and coworkers first performed Sanger sequencing to determine transposon insertion sites (12), and later, various versions of combining transposon mutagenesis and next-generation sequencing were developed, such as TraDIS (13), INSeq (14), HITS (15), Tn-seq (16) and Tn-seq Circle (17), here collectively referred to as Tn-seq. The application of Tn-seq has allowed for significant advancements in studies on essential genes over the past few years, resulting in (i) a dramatic increase in the number of prokaryotic species with gene essentiality screens; (ii) a revision of the essential-gene concept that includes all essential genomic elements, such as noncoding RNAs, rather than focusing on protein-coding genes only and (iii) gene essentiality screens in a wide array of experimental conditions *in vitro* and *in vivo*, rather than focusing only on rich media in cell culture.

We constructed a database of essential genes (DEG) in 2004 (18), and DEG 5.0 included essential genes of both bacteria and eukaryotes (19). In addition to DEG, other essential gene databases include EGGS (Essential Genes on Genome Scale, http://www.nmpdr.org/FIG/eggs.cgi) and OGEE (online gene essentiality database) (20), where the former hosts microbial gene essentiality data experimentally obtained from published genome-scale gene essentiality screens and the latter hosts essential-gene data obtained from large-scale experiments with associated gene features and text-mining results. Because of text-mining results, OGEE has most essential-gene records, while DEG entries are human curated and is the only one supporting BLAST searches. We have constructed DEG 10 to accommodate the quantitative and qualitative advancements in identifying essential genes by genome-wide essentiality screens in recent years, and the following is a summary of new database developments.

- In addition to protein-coding genes, DEG 10 now harbors essential genomic elements, including noncoding RNAs, promoters, regulatory sequences and replication origins (21,22).
- The number of bacteria with saturated genome-wide gene essentiality screens has nearly tripled, compared with that in DEG 5 (19).
- DEG 10 contains essential genomic elements determined not only *in vitro* (culture dishes), but also *in vivo* (intact mice) (14), not only for survival but also for pathogenesis (23), not only in rich media, but also in more diverse conditions, such as those required for cholesterol catabolism (24), antibiotic resistance (17), bile acid tolerance (13,25) and bacteriophage infection (26).
- DEG 10 hosts archaeal essential genes determined from the first gene essentiality screen in an archaeal genome (27).
- DEG 10 hosts both essential and nonessential protein-coding genes.
- DEG 10 is integrated with customizable BLAST tools that allow users to perform species- and experiment-specific searches for a single gene, a list of genes, annotated or unannotated genomes.

Therefore, DEG 10 (www.essentialgene.org) reflects the progress of the research on essential genes by including essential genomic elements under different conditions in three domains of life, with customizable BLAST tools.

## DATABASE NEW DEVELOPMENTS

### Increased number of bacterial species with genome-wide essentiality screens

The combination of high-throughput sequencing and high-density transposon mutagenesis has largely accelerated the process in determining essential genes. Compared to DEG 5 (19), the number of bacteria with saturated genome-wide gene essentiality screens has nearly tripled in DEG 10, which has data for 31 bacteria. DEG 10 contains more than 12 000 bacterial essential genes, more than twice the number of those in DEG 5. The figures corresponding to newly added essential genes are highlighted in Table 1.

**Table 1.** Contents in DEG 10

Domain of life	Organism	No. of essential genomic elements				
		Coding	Noncoding	Method^a^	Saturated	Ref.
Bacteria	*Acinetobacter baylyi*	**499**		Single-gene knockout	Yes	(50)
	*Bacillus subtilis*	261	**2**	Single-gene knockout	Yes	(6,51)
	*Bacteroides thetaiotaomicron*	**325**		INSeq	Yes	(14)
	*Burkholderia thailandensis*	**406**		Tn-seq	Yes	(52)
	*Campylobacter jejuni*	**233**		Transposon mutagenesis	Yes	(53)
	*Caulobacter crescentus*	**480**	**532**	Tn-seq	Yes	(22)
	*Escherichia coli*	620		Genetic footprinting	Yes	(54)
	*Escherichia coli*	303		Single-gene knockout	Yes	(7)
	*Francisella novicida*	**396**		Tn-seq	Yes	(55)
	*Haemophilus influenzae*	667		Genetic footprinting	Yes	(56)
	*Helicobacter pylori*	344		Transposon mutagenesis followed by by microarray (MATT)	Yes	(57)
	*Mycobacterium tuberculosis*	614		Transposon mutagenesis followed by hybridization (TraSH)	Yes	(58)
	*Mycobacterium tuberculosis*	**774**		Tn-seq	Yes	(24)
	*Mycobacterium tuberculosis*	**742**	**35**	Tn-seq	Yes	(21)
	*Mycoplasma genitalium*	382		Transposon mutagenesis followed by Sanger sequencing, Single-gene knockout	Yes	(12,59)
	*Mycoplasma pulmonis*	**321**		Transposon mutagenesis followed by Sanger sequencing	Yes	(60)
	*Porphyromonasgingivalis*	**463**		Tn-seq	Yes	(42)
	*Pseudomonas aeruginosa*	335		Transposon mutagenesis followed by genetic footprinting	Yes	(61)
	*Pseudomonas aeruginosa*	**117**		Tn-seq	Yes	(17)
	*Salmonella entericaserovar Typhi*	**356**		TraDIS	Yes	(13)
	*Salmonella enterica Typhimurium*	**306**	**15**	TraDIS	Yes	(29)
	*Salmonella entericaserovar Typhimurium*	**105**		Tn-seq	Yes	(25)
	*Salmonella entericaserovar Typhimurium SL1344*	**353**	**23**	TraDIS	Yes	(29)
	*Salmonella entericaserovar Typhi Ty2*	**358**	**24**	TraDIS	Yes	(29)
	*Salmonella typhimurium*	490		Insertion-duplication	Yes	(62)
	*Shewanella oneidensis*	**403**		Transposon mutagenesis followed by microarray	Yes	(63)
	*Sphingomonas wittichii*	**579**	**32**	Tn-seq	Yes	(64)
	*Staphylococcus aureus*	302		Antisense RNA; Allelic Replacement Mutagenesis	No	(65-67)
	*Staphylococcus aureus*	**351**		Transposon-Mediated Differential Hybridisation (TMDH)	Yes	(68)
	*Streptococcus pneumoniae*	244		Insertion-duplication, allelic replacement mutagenesis	No	(69,70)
	*Streptococcus pneumoniae*		**72**	Tn-seq	Yes	(23)
	*Streptococcus sanguinis*	**218**		Single-gene knockout	Yes	(41)
	*Vibrio cholerae*	**789**		Tn-seq	Yes	(71)
Archaea	*Methanococcus maripaludis*	**519**		Tn-seq	Yes	(27)
Eukaryotes	*Arabidopsis thaliana*	358		Single-gene knockout	No	(48)
	*Aspergillus fumigatus*	**35**		Conditional promoter replacement	No	(72)
	*Caenorhabditis elegans*	294		RNA interference	No	(73)
	*Daniorerio*	315		Insertional mutagenesis	No	(74)
	*Drosophila melanogaster*	376		P-element insertion	No	(75)
	*Homo sapiens*	2452		Orthologs and literature search	No	(76,77)
	*Mus musculus*	2136		Single-gene knockout	No	(78)
	*Saccharomyces cerevisiae*	1110		Single-gene knockout	Yes	(37)
	*Schizosaccharomyces pombe*	**1260**		Single-gene knockout	Yes	(38)

Figures in bold denote newly added essential genes.

^a^Genetic footprinting is a method that performs transposon mutagenesis followed by PCR to determine transposon insertion sites (79). Tn-seq here collectively refers to a method that uses the next-generation sequencing to determine transposon insertion sites, including, TraDIS, INSeq, HITS, Tn-seq and Tn-seq Circle.

In addition to essential genes, in fact, nonessential genes can be determined as well in most genome-wide essentiality screens. Single-gene knockout experiments directly determine whether a particular gene is essential or nonessential. Genome-wide transposon mutagenesis determines nonessential genes first, because all recovered mutants only harbor transposon insertions in nonessential genes, while essential genes are, in fact, inferred. Therefore, nonessential genes can be reliably identified by both kinds of approaches. Because information about nonessential genes can be important as well, DEG 10 hosts nonessential genes, which are organized into a sub-database.

### Determination of essential noncoding genomic elements

It is increasingly being recognized that bacterial genomes encode large amounts of noncoding RNAs (28). The use of high-density transposon mutagenesis and high-throughput sequencing makes identification of essential noncoding RNAs possible. In the genome of *Caulobacter crescentus*, 428 735 unique Tn5 insertions were generated and mapped in its 4 Mb genome. Therefore, in addition to identifying 480 essential protein-coding genes, 29 tRNAs and eight small noncoding RNAs were also found to be essential (22). In *Mycobacterum tuberculosis*, 36 488 transposon insertions were generated and mapped, and in addition to essential protein-coding genes, 25 nondisruptable genomic segments were found. These segments include 10 tRNAs and the RNA catalytic unit of RNaseP, which is required for tRNA processing (21). In a study with a similar method for the *Salmonella serovars Typhimurium*, 15 noncoding RNAs were found to be essential (29). It is noteworthy that RNaseP was again among the identified essential noncoding RNA, and therefore it is likely to be a widely required noncoding RNA among bacteria.

Mann *et al.* tested the hypothesis that some noncoding RNAs have niche-specific roles in virulence (23). Because increasing evidence suggests sRNAs are involved in pathogenesis, Mann *et al.* first performed RNA-seq to define the sRNA repertoire of *S. pneumonia*, a causative agent for pneumonia, and identified 89 sRNAs. To examine organ-specific roles in pneumococcal pathogenesis, they generated a pool of pneumococcal mutants by transposon mutagenesis, administrated the mutants in organs vital to the progression of pneumococcal diseases, the nasopharynx, lungs and bloodstream, and performed deep sequencing in DNA from recovered mutants. Consequently, 28 sRNAs in the lung, 26 in the nasopharynxand 18 in the blood were found to alter fitness in these host niches. Therefore, this study used Tn-seq to assay the role of sRNA in pathogenesis in a niche-specific manner (23).

In addition to noncoding RNAs, other noncoding elements of the genome can be essential as well. These include promoters of some essential protein-coding genes, regulatory sequences and replication origins. Indeed, Christen *et al.* identified 402 essential promoter regions and two essential elements in the replication origin of the Caulobacter genome, in addition to 91 essential intergenic sequences with unknown functions (22). Zhang *et al.* identified 35 intergenic elements for optimal growth of *M. tuberculosis* (21). DEG 10 collects the above identified noncoding genomic elements, with annotations from the Rfam database (30), if relevant annotations are available. Because of the apparent essential role of replication origins, DEG 10 also links to DoriC, which is a database of bacterial and archaeal replication origins (31).

### Determination of essential genes under diverse conditions

The application of high-throughput sequencing makes it possible to determine and quantify contributions of essential genes to organism fitness under conditions that are not practical by using other methods, because of the digital nature of the next-generation sequencing. Therefore, in addition to regular rich medium in cell cultures, in the past several years, bacterial essential genes have been identified under a large number of different conditions, e.g. in intact mice.

One illustrative example is the study on genes required to establish a human gut symbiont (14). Goodman *et al.* first performed the INseq method (transposon mutagenesis followed by next generation sequencing) to identify a set of essential genes for the commensal *B. thetaiotaomicron in vitro*. Next, they examined the genes critical for fitness *in vivo*, i.e. in a mammalian gut ecosystem by colonizing bacterial mutants in germ-free mice. By comparing the input (before inoculation) and output (recovered bacteria), 280 genes showed underrepresentation, suggesting them to be critical for *in vivo* fitness. By changing the experimental conditions such as in the presence of human gut-associated bacteria, they identified five adjacent genes that conferred fitness disadvantages during monoassociation of germ-free mice, while showing no impact on bacterial growth *in vitro*, thus highlighting the importance of the *in vivo* context in determining gene essentiality (14).

In a large-scale study, van Opijnen and Camilli performed Tn-seq on the genome of *S. pneumonia* under 17 *in vitro* conditions (e.g. pH, temperature, antibiotic, heavy metal, stress, nutritional stimulation) and two *in vivo* conditions (carriage and infection), and have identified over 1,800 genotype-phenotype genetic interactions and associated pathways (32). Other condition-specific studies include the identification of essential genes for bile acid tolerance, a trait required of an enteric bacterium and for carriage of *S. Typhi* in the gall bladder (13,25), resistance to the aminoglycoside antibiotic tobramycin in *Pseudomonas aeruginosa* (17), bacteriophage infection of *S. Typhi* to assess for Vi polysaccharide capsule expression (26) and cholesterol metabolism in *M. tuberculosis* (24). In addition to those determined in rich medium only, DEG 10 harbors condition-specific essential genes as well.

### Determination of essential genes in an archaeal genome

Archaea are prokaryotes that constitute a separate domain of life, in addition to bacteria and eukaryotes (33). Some archaea can survive in extreme conditions, such as highly salty or hot environments. Methanogenesis, a process to generate methane, is a specialized anaerobic respiration that requires distinctive biochemical reactions unique to methanogenic archaea, which are responsible for 80% of the methane in greenhouse gas (34).

By using the method of Tn-seq, Sarmiento *et al.* identified essential genes in hydrogenotrophic, methanogenic archaeon *Methanococcusmaripaludis* S2, and this was the first genome-wide gene essentiality screen in archaea (27). About 89 000 unique transposon inserts were mapped, and 526 genes were classified as essential in rich medium. Similar to bacteria, many essential genes encode fundamental cellular processes, such as transcription, translation and replication. Some essential genes, however, are unique to the archaeal or methanococcal lineages. For instance, the DNA polymerase PolD is essential, whereas the archaeal homolog of bacterial PolB is not (27).

### Determination of essential eukaryotic genes

In contrast to prokaryotic essential genes, which have had a dramatic increase in past years, the number of eukaryotic essential genes, while climbing steadily, does not exhibit a drastic increase, apparently due to the lack of genome-wide mutagenesis strategies. To generate single-gene knockout, however, takes much more effort, and therefore usually requires multi-center collaborations. The aim of the International Knockout Mouse Consortium (IKMC), formed in 2007, is to generate mutant mouse lines with all genes deleted one by one (35). A recent report showed mouse gene deletion mutants have been obtained for ∼17 000 of the total 20 000 protein-coding genes (36). Therefore, in the near future, we expect to have a complete set of essential mouse protein-coding genes. With *Saccharomyces cerevisiae* being the first eukaryote to have all of its single-gene deletion mutants generated (37), DEG 10 has added essential genes of *Schizosaccharomyces pombe*, which is the second eukaryote that has a saturated gene deletion study (38).

### Customizable BLAST tools

Performing homologous searches with the BLAST program (39) against DEG is common (40–43), and therefore to facilitate this use, we have developed a set of customizable BLAST tools. Users have the following four options.

- To perform BLAST search for a single gene. The major improvement for this option is that users now can perform species-specific BLAST search, in addition to having the option to change *P*-values. The output is unprocessed BLAST raw results.
- To perform BLAST search for a list of genes. Users can submit a list of protein or DNA sequences, and the BLAST output will be organized and processed to generate an XML file that is parsed by the Biopython module (44). The output includes how many genes among the queried gene set have DEG homologs, and how many homologous genes in DEG are found. All homologous genes are clickable by linking to corresponding alignments. The above function can also be done in a species-specific manner.
- To perform BLAST search for annotated genome sequences. Because of the increasing pace of genome sequencing, in many cases users need to analyze whole-genome sequences. By using this option, users can submit a whole-genome sequence or scaffold, with annotation information, i.e. either in the GenBank format or by uploading Protein Table Files (PTT format).
- To perform BLAST search for unannotated genome sequences. If users need to analyze whole-genome sequences that have not been annotated, DEG is integrated with two gene-finding programs, Zcurve (45) and Glimmer (46), for gene identification. Protein-coding genes are first identified by Zcurve or Glimmer, and then BLAST searches are performed against DEG. For both Options 3 and 4, the output is processed and organized to convey information on the number of homologs in DEG and in queried genomes, with linking to alignments. The XML files and resulting webpages are stored for 7 days on the server, and can be retrieved as needed.

With the aforementioned new tools, users can perform BLAST searches for single genes, multiple genes, annotated genomes or unannotated genomes with filters to restrict the search to a subset of species or experiments with desirable *P*-values.

## FUTURE PERSPECTIVE

Recent breakthroughs in sequencing technology, i.e. the next-generation sequencing that parallelizes the process to sequence millions of reads concurrently, have fundamentally changed many areas of biological research, and the research on essential genes is no exception. Significant advancements have been made in essential-gene studies, for example, the concept of the essential gene has been revised to include all essential genomic elements, rather than focusing on protein-coding genes only. It is not difficult to envision that in the near future, genome-wide gene essentiality screens will be performed in a large number of bacteria and archaea, under increasingly diverse experimental conditions, and will result in dramatic increases in identified prokaryotic essential genomic elements. The accumulation of essential-gene information will be particular helpful in identifying bacterial drug targets (47) and in constructing the minimal genome in studies of synthetic biology (4). Without breakthroughs in genome-wide mutagenesis technology, however, there will likely be no dramatic increases in identified eukaryotic essential genes. Nevertheless, it is expected that single-gene knockout projects for the model organisms, such as mice and *Arabidopsis thaliana* (48), will soon be completed. It is increasingly being recognized that mammalian genomes have highly complex transcriptomes (49), and therefore we would expect that some eukaryotic noncoding elements, such as long noncoding RNAs, will be identified as essential. DEG will continue to incorporate newly discovered essential genomic elements in a timely manner to keep pace with this rapidly developing field.

## FUNDING

Startup fund from Wayne State University (to R.Z.). Funding for open access charge: In part by the National Natural Science Foundation of China [31171238, 30800642 and 31200991]; Program for New Century Excellent Talents in University [No. NCET-12-0396].

*Conflict of interest statement.* None declared.
